# Secretion of *Clostridium difficile* Toxins A and B Requires the Holin-like Protein TcdE

**DOI:** 10.1371/journal.ppat.1002727

**Published:** 2012-06-07

**Authors:** Revathi Govind, Bruno Dupuy

**Affiliations:** 1 Laboratoire Pathogenèse des Bactéries Anaérobies, Institut Pasteur, Paris, France; 2 Division of Biology, Kansas State University, Manhattan, Kansas, United States of America; Dartmouth Medical School, United States of America

## Abstract

The pathogenesis of *Clostridium difficile*, the major cause of antibiotic-associated diarrhea, is mainly associated with the production and activities of two major toxins. In many bacteria, toxins are released into the extracellular environment via the general secretion pathways. *C. difficile* toxins A and B have no export signature and their secretion is not explainable by cell lysis, suggesting that they might be secreted by an unusual mechanism. The TcdE protein encoded within the *C. difficile* pathogenicity locus (PaLoc) has predicted structural features similar to those of bacteriophage holin proteins. During many types of phage infection, host lysis is driven by an endolysin that crosses the cytoplasmic membrane through a pore formed by holin oligomerization. We demonstrated that TcdE has a holin-like activity by functionally complementing a λ phage deprived of its holin. Similar to λ holin, TcdE expressed in *Escherichia coli* and *C. difficile* formed oligomers in the cytoplamic membrane. A *C. difficile tcdE* mutant strain grew at the same rate as the wild-type strain, but accumulated a dramatically reduced amount of toxin proteins in the medium. However, the complemented *tcdE* mutant released the toxins efficiently. There was no difference in the abundance of *tcdA* and *tcdB* transcripts or of several cytoplasmic proteins in the mutant and the wild-type strains. In addition, TcdE did not overtly affect membrane integrity of *C. difficile* in the presence of TcdA/TcdB. Thus, TcdE acts as a holin-like protein to facilitate the release of *C. difficile* toxins to the extracellular environment, but, unlike the phage holins, does not cause the non-specific release of cytosolic contents. TcdE appears to be the first example of a bacterial protein that releases toxins into the environment by a phage-like system.

## Author Summary

*Clostridium difficile* is the causative agent of antibiotic associated diarrhea and has become the most prevalent cause of infectious nosocomial diarrhea in North America and in several countries in Europe. Most virulent strains of *C. difficile* produce two high molecular weight toxins that are regarded as the primary virulence factors. The mechanism by which these large toxins are secreted from bacterial cells is not known. Unlike most clostridial toxins, they have no export signature and must be secreted by an unusual system. This work investigated the role of a *C. difficile* membrane protein TcdE in the release of toxins from the bacterial cell. We showed that *C. difficile tcdE* mutants were defective in toxin release and present evidence that *C. difficile* TcdE protein activity is similar to that of bacteriophage holin proteins required for lysis of host cells after intracellular phage development. These results suggest that TcdE helps efficient secretion of toxins by a phage type system. However, unlike phages, TcdE does not induce cell lysis. A detailed, mechanistic understanding of the holin-dependent system that mediates toxin secretion may helpful for the development of strategies for preventing and treating *C. difficile* infections.

## Introduction

*Clostridium difficile*, a Gram-positive, anaerobic bacterium, is a major cause of antibiotic-associated diarrhea and pseudomembraneous colitis. *C. difficile* infections (CDI) are typically induced by treatment with antibiotics that disrupt the normal gastrointestinal microbiota. *C. difficile* has emerged in the last decade as a formidable enteric pathogen with an increased propensity to cause frequent, severe and recurrent disease [1], [2]. This mainly results from the emergence of new isolates, such as those that have been assigned to the BI/NAP1/027 family, which was first reported in North America and has rapidly spread among various European countries. Pathogenic *C. difficile* strains usually produce two high molecular weight toxins, TcdB and TcdA, that are the major virulence factors [3]. Both toxins are monoglucosyltransferases that modify the activity of members of host cell Rho family of small GTPases [4], disrupting the actin cytoskeleton of intestinal epithelial cells. TcdA (308 kDa) and TcdB (270 kDa) are among the largest bacterial toxins reported to date. They share 49% amino acid identity and a similar overall structure composed of a receptor-binding domain, a transmembrane domain and a glycosyl transferase domain [5], [6]. Important advances have been made in understanding the regulation of toxin synthesis, their enzymatic activities and their impact on host cell physiology [4], [7]–[12], but their mode of secretion from the bacteria has been a mystery. Most extracellular proteins carry an N-terminal or C-terminal signal peptide, a Tat-signal peptide or some other clearly definable secretion signal [13]. Interestingly, TcdA and TcdB are secreted without any apparent signal peptide or other recognizable secretion signal. Moreover, bacterial lysis does not seem to explain release of toxin proteins, since Karlsson *et al* [14] showed that in stationary phase cells 50% of the total toxin protein synthesized is released into the extracellular fluid whereas <1% of typical cytoplasmic proteins is released.

**Figure 1:**
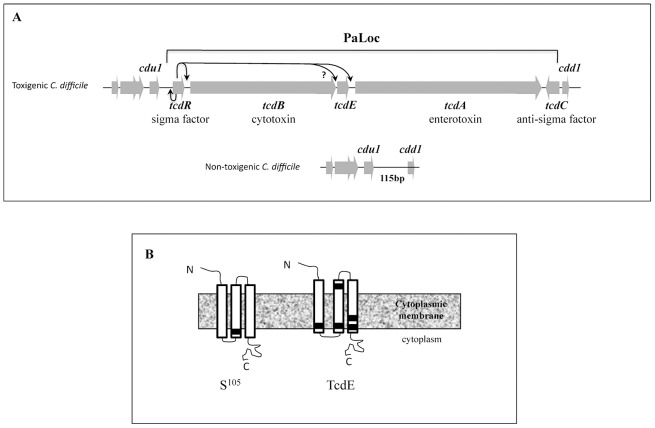
PaLoc in *C. difficile*. **A.** Schematic diagram of PaLoc in toxigenic *C. difficile* strains. In non-toxigenic strains this region is replaced by a short 115 bp sequence. Arrows indicates the positive regulation of *tcdR*, *tcdB* and *tcdA* by σ^TcdR^. **B** Predicted topology of TcdE and. λS holin Horizontal black bars represent the relative position of Cys 51 in S^105^ and Cys 29, 56, 79, 111 and 113 in TcdE.

The toxin genes *tcdA* and *tcdB* lie within a chromosomal region of 19.6 kb designated the pathogenicity locus (PaLoc) (Figure 1A). The same locus encodes TcdR, an alternative sigma factor that specifically directs transcription from the toxin promoters as well as its own promoter, TcdC, an antagonist of TcdR that prevents the formation of the TcdR-containing RNA polymerase holoenzyme [12], [15], [16], and TcdE, whose function was not known.

When overexpressed in *Escherichia coli*, TcdE causes cell death [17]. The *tcdE* open reading frame potentially encodes a small, hydrophobic protein of 166 amino acids with a short hydrophilic stretch at the N-terminus and a series of charged residues at the C-terminus [17]. TcdE is predicted to contain three transmembrane domains (Figure 1B). These structural features and primary sequence similarities strongly suggest that TcdE is a member of the class I holins of which phage λ S protein is a member. Holins are small membrane proteins encoded by double-stranded DNA phages that are required for the lysis of host cells at a programmed time after completion of intracellular phage development [18], [19]. They form disruptive lesions by oligomerization in the host cell plasma membrane to allow a prophage-encoded endolysin (a muralytic enzyme), to cross the membrane and attack the murein, resulting in cell lysis and release of phage particles [18], [19]. Phage λ S protein is made in two forms. S^105^ has holin activity, whereas S^107^, which has two additional amino acid residues at the N-terminus, retards lysis by interfering with the activity of S^105^.

While most holins are associated with terminal lysis of phage-infected bacteria, some holin-like proteins were also suggested to be responsible for the release of proteins from uninfected bacteria [20]–[23]. However this supposition has never been tested experimentally and the role of holin-like proteins in protein secretion has yet to be proven. The homology of TcdE to phage holins led us to investigate its possible role in toxin secretion.

In this study, we first showed that TcdE is required for efficient secretion of toxins. Surprisingly, TcdE facilitates release of toxins without inducing cell lysis or general membrane permeability. We then demonstrated that TcdE has a holin-like activity by complementing an *E. coli* λ lysogen that is defective for the λ holin. Several models for the TcdE-dependent secretion of TcdA/TcdB are suggested taking account of the absence of cell lysis due to TcdE holin activity in the natural host. These results provide the first experimental evidence that a holin-like protein has a role in protein secretion.

## Results

### TcdE is required for efficient secretion of toxins

**Figure 2:**
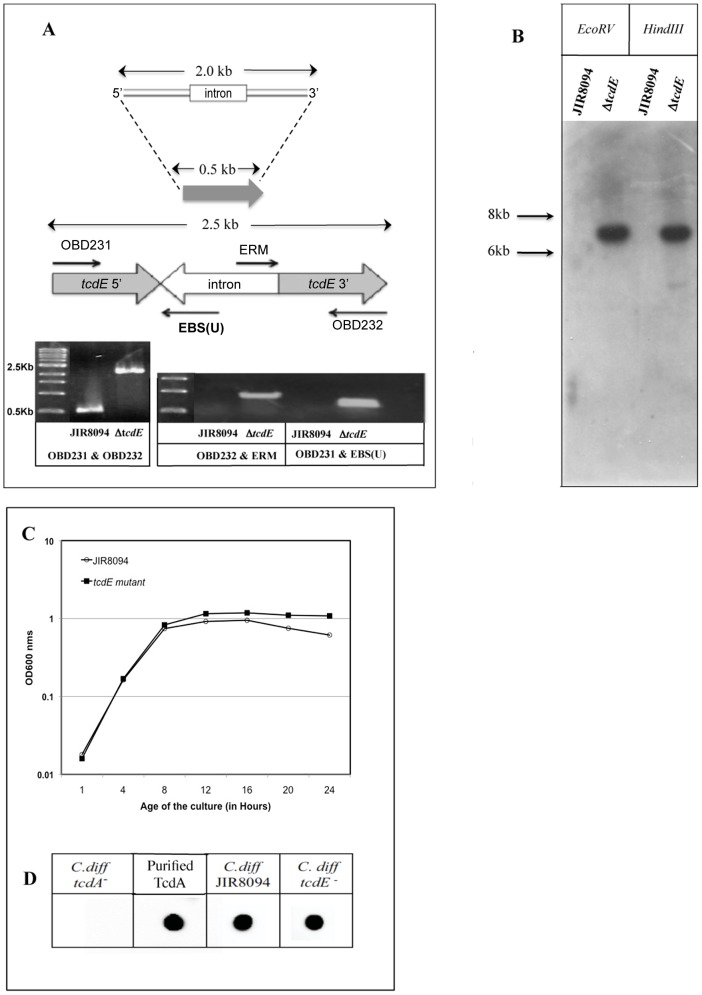
Construction and characterization of the *tcdE* mutant in *C. difficile*. **A.** PCR verification of the intron insertion using gene-specific primers OBD231 and OBD232 or the intron-specific primer EBS(U) or ERM in association with primers OBD231 or OBD232. **B.** Southern blot analysis of genomic DNA from *C. difficile* JIR8094 and *tcdE* mutant strains with an intron probe. Chromosomal DNA was digested by either *Eco*RV or *Hin*dIII. **C.** Growth curves of JIR8094 and *tcdE* mutant in TY medium. **D.** TcdA Dot blot analysis. The crude lysates prepared by sonication of cells with their supernatants (200 ng proteins) were probed with TcdA monoclonal antibody (PCG4).

**Figure 3:**
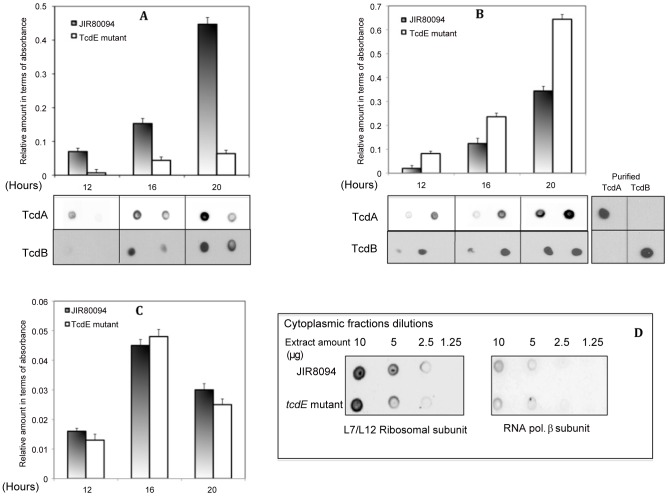
Quantification of toxins and lactate dehydrogenase (LDH) activity in parent JIR8094 and *tcdE* mutant strains. Toxin titers in the culture supernatants (**A**) and in cytoplasmic proteins (**B**) were determined by ELISA and the signal from the test was recorded as absorbance at 450 nm. The data shown are the mean +/− standard error of three replicative samples. Student's *t*-test was used for statistical analysis. (*) *P*-value is < to 0.05. Dot blots with monoclonal anti-TcdA and anti-TcdB are shown in the lower panels. Purified TcdA and TcdB were used as controls. **C.** LDH activity in the cytoplasmic fraction was determined using Promega CytoTox 96 and was measured as micro units calculated in comparison with standards provided in the kit. **D.** Cytoplasmic proteins collected from a 16 hours old cultures of parental and *tcdE* mutant strains were analyzed in dot blots using monoclonal antibodies against L7/L12 ribosomal subunits and the RNA polymerase beta subunit.

We used the ClosTron system recently developed by Heap and coworkers [24] to disrupt the *tcdE* gene by insertion of a group II intron from pMTL007 (see Materials and Methods). To confirm the disruption, the *tcdE*-specific primers OBD231 and OBD232 were used to amplify a 550 bp PCR product from the parental JIR8094 DNA, corresponding to the wild type *tcdE* gene, and a 2.5 kbp product from the mutant DNA, corresponding to the *tcdE* gene with group II intron inserted within it (Figure 2A). When PCR was carried out using intron-specific primers EBS(U) and ERM along with OBD232 and OBD231, PCR products of 1150 bp and 570 bp respectively could be amplified from *tcdE* mutant DNA but not from the wild-type strain's DNA (Figure 2A) confirming the insertion of the intron in the *tcdE* gene. Furthermore, Southern blot analysis confirmed that the intron had inserted in only one DNA region in the *tcdE* mutant (Figure 2B). The wild-type and the *tcdE* mutant strains grew similarly in TY broth, but a slightly higher OD_600_ was seen for the mutant strain compared the wild-type after 16 hrs (Figure 2C). In quantitative reverse transcription-PCR assays the levels of *tcdA* and *tcdB* transcripts were the same for the parental and mutant strains (data not shown). This result suggests that intron insertion within *tcdE* exerts no polar effect on the expression of *tcdA*, consistent with the fact that *tcdA* transcription occurs primarily from its own promoter (Figure 1A) [12]. We confirmed by dot blot experiments that the quantity of TcdA detected in total crude extracts of both strains was the same (Figure 2D). However, when we analyzed the culture supernatants, we observed a dramatic reduction in the amount of toxin secreted by the mutant when compared to the wild-type strain (Figure 3A). On the other hand, more toxin accumulated in the cytoplasm of the *tcdE* mutant than in the wild-type strain in stationary phase cells (Figure 3B). Both observations were confirmed by dot blot analysis with monoclonal antibodies against TcdA and TcdB (Figure 3AB) and Vero cell cytotoxicity assays, which is predominantly toxin B-assessed (Figure S1).

**Figure 4:**
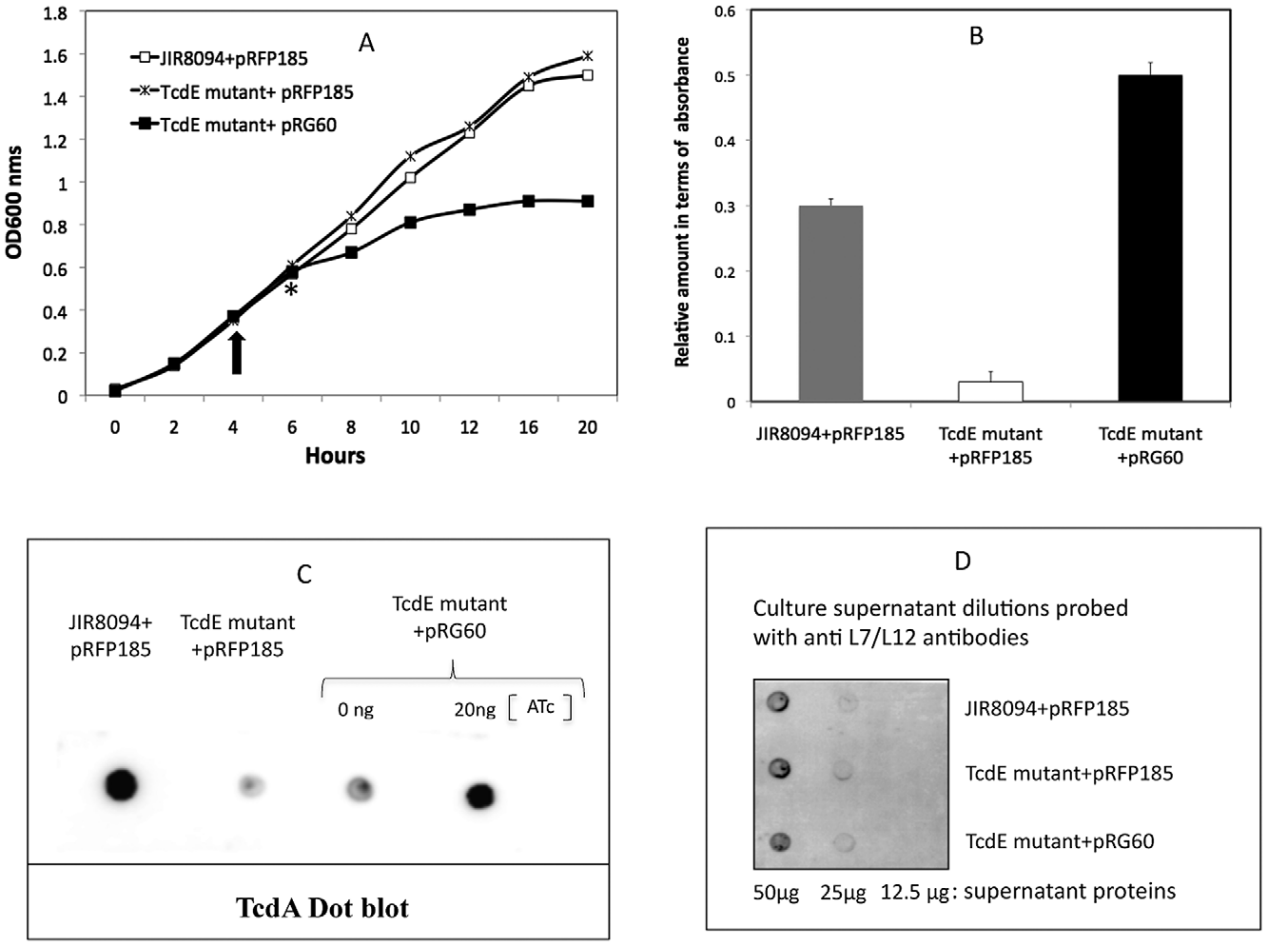
Complementation of TcdE mutant. **A** Growth curve of parent, the TcdE mutant and the complemented TcdE mutant strains. A. The inducer ATc (20 ng/ml) was added to bacterial cultures at 4 hrs after inoculation, indicated by an arrow. The star * indicates the time point when the cultures were harvested for toxin release analysis. **B.** Toxins were quantified by ELISA from supernatants of bacterial cultures induced by 20 ng/ml ATc for 2 hours. The signal from the test was recorded as absorbance at 450 nm. The data shown are the mean +/− standard error of three replicative samples. **C.** Dot blots of culture supernatants of the parental, the TcdE mutant and the complemented TcdE mutant, induced or not induced by ATc (0 and 20 ng/ml), with monoclonal anti-TcdA. **D.** Dot blots of samples in B with monoclonal antibodies against L7/L12 ribosomal subunits.

To confirm that the defect in toxin secretion was due to the disruption of TcdE, we complemented the *tcdE* mutant with the wild type *tcdE* gene. Expression of TcdE from its own promoter using a multicopy plasmid was observed to be lethal to *C. difficile* (see below). Hence we expressed TcdE in the mutant strain using a tightly controlled expression system [25]. The *tcdE* ORF with a C-terminal 6xHis Tag was cloned downstream of a tetracycline-inducible promoter in the vector pRPF185 to create pRG60 (see Materials and Methods). We first showed that TcdE-6His is expressed in *C. difficile* cultures induced with 20 ng/ml to 50 ng/ml of ATc (Anhydrotetracycline), a non-antibiotic analog of tetracycline, without affecting cell growth (data not shown). Thus, we induced the *C. difficile* cultures with ATc (20 ng/ml) to test the effect of TcdE-6His on toxin release in the complemented mutant strain. The *tcdE* mutant carrying pRG60 along with the control strains JIR8094 and the *tcdE* mutant carrying the vector pRPF185 were grown for 4 hours to an OD600 of 0.3 in TY broth and induced with ATc. No difference in growth of the three strains could be observed during the first two hours after induction (Figure 4A). However, after more than two hours, the growth rate of the complemented *tcdE* mutant began to decrease (Figure 4A). Hence, using ELISA, we measured toxin proteins in the supernatant fluid of cultures two hours after induction with ATc. The culture supernatant of the complemented *tcdE* mutant had a higher concentration of toxin proteins than did the parent strain JIR8094 or the *tcdE* mutant (Figure 4B). We confirmed by a dot blot experiment with monoclonal antibodies against TcdA that the *tcdE* gene on pRG60 complements the *tcdE* mutant (Figure 4C). Thus, these data imply that TcdE is directly required for toxin release.

### TcdE-dependent release of toxin is not related to bacterial cell lysis

**Figure 5:**
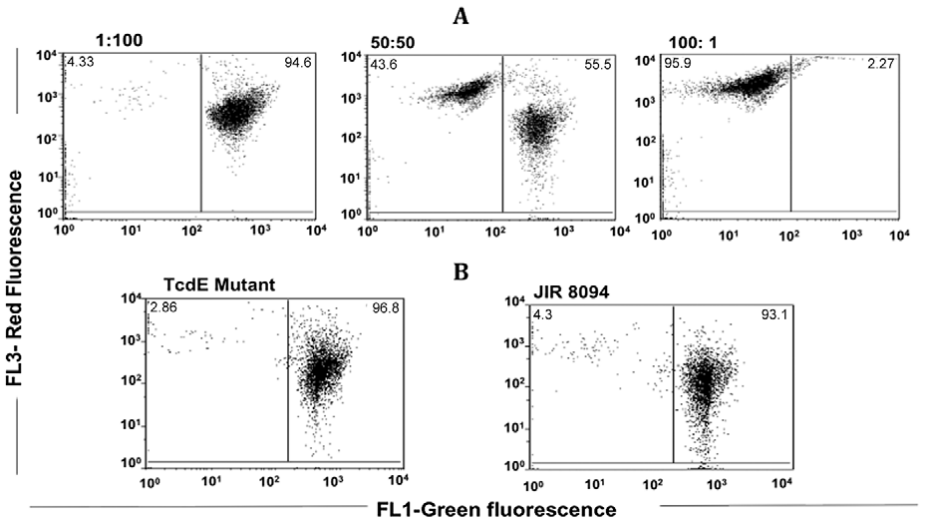
FACS analysis of *C. difficile* cells for membrane permeability through propidium iodide (PI) and SYTO staining. **A.** The viability standard samples containing the heat killed and actively growing *C. difficile* cells at 1/100, 50/50 and 100/1 ratio, respectively. **B.** The *tcdE* mutant and the parent JIR8094 cells collected from the overnight (16 h) cultures, were subjected to FACS analysis following propidium iodide (PI) and SYTO staining.

**Figure 6:**
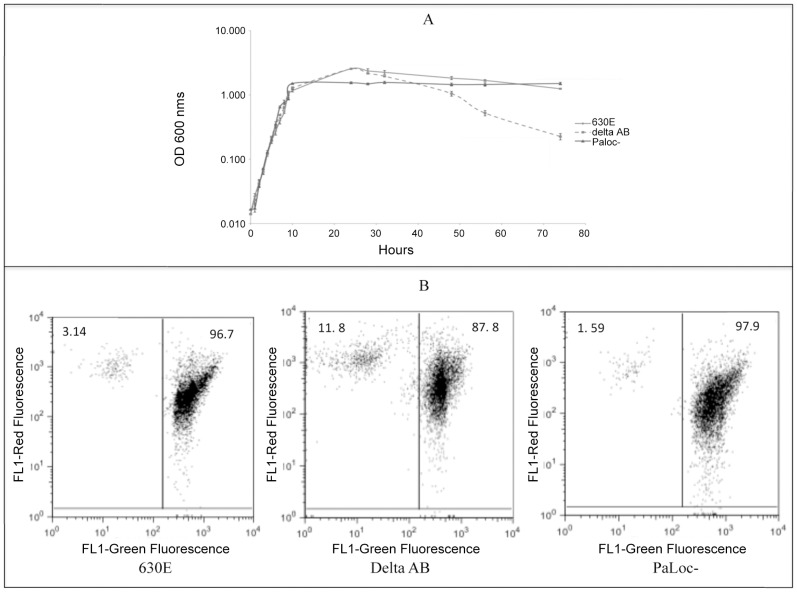
Cell lysis in *C. difficile* A^−^B^−^ double mutant strain. **A.** Growth curves of strains, 630A^−^B^−^ (*C. difficile tcd*AB double mutant), 630E (wild type) and a PaLoc negative strain. *C. difficile* strains were grown in TY medium in a 100 ml Erlenmeyer flask and the optical density at 600 nms was recorded at regular time interval. **B.** Bacterial cultures were harvested at a 30 hour time point for FACS analysis after propidium iodide (PI) and SYTO staining.

To test whether TcdE mediates toxin release via cell lysis, we compared the levels of several known cytosolic marker proteins in the *tcdE* mutant and wild-type strains. No significant difference was seen between the parent strain and the mutant in their levels of cytosolic LDH activity (Figure 3C). In addition, dot blots of cytosolic fractions of overnight cultures (16 hrs) with antibodies to ribosomal subunits (L7/L12) and the RNA polymerase β subunit (see Materials and Methods) showed similar levels of these proteins in the cytosol of the mutant and wild-type strains (Figure 3D). In the complemented *tcdE* mutant, the amounts of L7/L12 proteins in the culture supernatants were very low and equal to those detected for the parental and *tcdE* mutant strains (Figure 4D). Finally, we looked for an effect of TcdE expression on *C. difficile* membrane integrity. We used FACS analysis of cells exposed to the fluorescent nucleotide binding dyes SYTO9 and propidium iodide (PI). SYTO9 is commonly used as a stain for live cells, whereas PI is excluded by the intact cell membrane, thus staining a cell only when the integrity of the membrane has been compromised. When used in combination, cells with intact membranes are labeled green by SYTO9 while membrane-permeablized cells are labeled red by PI. FACS analysis of control samples, containing mixtures of heat-killed and actively growing *C. difficile* cells, was consistent with the different ratios (1/100, 50/50 and 100/1 of killed/live, respectively) that we used (Figure 5A). When we analysed the mutant and parental strains harvested 16 hrs after inoculation, no significant difference could be observed in the intact vs. membrane-permeable cell populations (Figure 5B). In both strains, the membrane-permeable fraction was negligible indicating minimal cell lysis. Interestingly, cell lysis did occur in a *C. difficile tcdA tcdB* double mutant strain [26] that produces TcdE, as shown by the loss of absorbance at 600 nm (Figure 6A) and the appearance of red fluorescence (Figure 6B) when compared to the parental and PaLoc negative strains (see the Discussion).

### TcdE can function as a phage holin

**Figure 7:**
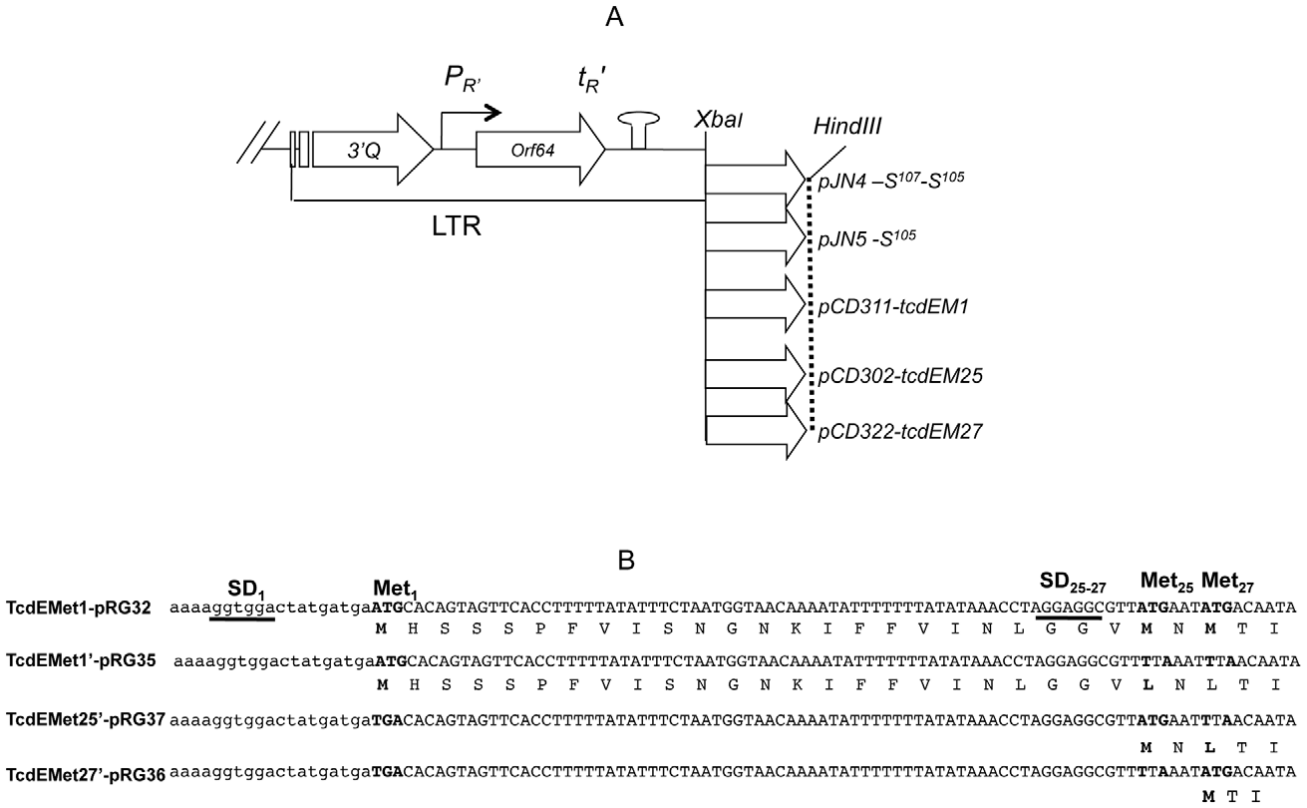
The holin like TcdE. **A.** Constructs used for holin expression under the control of the late transcription regulatory (LTR) elements of phage λ. The promoter *p_R′_* and the transcriptional terminator *t_R′_* of the λ LTR region are depicted as a bent arrow and a hairpin structure, respectively. **B.** TcdE sequence: all possible translational starts are indicated as Met_1_, Met_25_ and Met_27_, the potential Shine-Dalgarno sequences are underlined and mutated nucleotides in the specified constructs are highlighted in bold.

**Figure 8:**
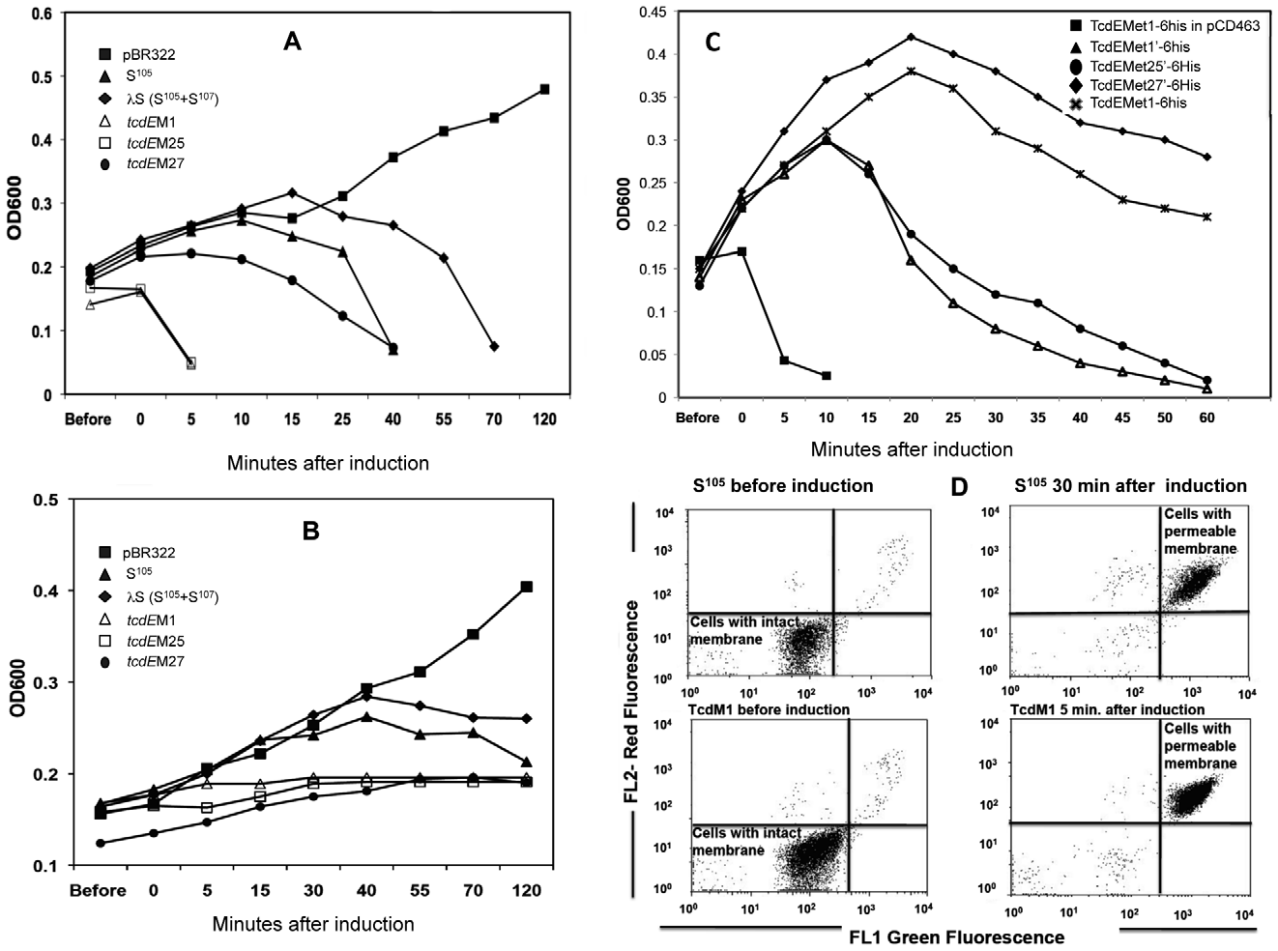
Testing holin function of TcdE in *E. coli*. Lysis curves of lysogenic cultures carrying λcI_857_ *Sam7* (**A**) or λCmrΔ(SR) (**B**) and plasmids expressing in trans λ*S*, *S^105^*,TcdEM1, TcdEM25, and TcdEM27, respectively. A lysogenic strain carrying pBR322 was used as negative control. **C.** Lysis curve of *E. coli* λcI_857_ *Sam7* lysogen carrying plasmids expressing TcdEM1 in pCD463, TcdEMet1, TcdEMet1', TcdEMet25' and TcdEMet27' respectively. **D.** FACS analysis of *E. coli* λCmrΔ(SR) lysogens expressing either S^105^ or TcdEM1 through propidium idodide (PI) and SYTO9 staining.

Since no bona fide system for assaying holin activity exists in *C. difficile*, we turned to assays of *E. coli* λ lysogens expressing TcdE. To test whether TcdE has any holin-like activity, we asked whether TcdE could complement a λ lysogen that has a nonsense mutation (Sam7) in its holin gene but has a functional endolysin gene [27]. We confirmed the functionality of this system using plasmids pJN4 and pJN5 (Figure 7A) expressing, respectively, a μιξτυρε of holin S^105^ and antiholin S^107^ or S^105^ alone from the λ late promoter and the λS RBS [28]. As shown in Figure 8A, thermo-induction of *E. coli* MC1063 λ (cI_857_Sam7) containing pJN4 or pJN5 led to complete bacterial lysis after 40 or 70 minutes, respectively, which is in good agreement with published values [18]. No lysis was observed after heat induction of a plasmid-free lysogen or a lysogen carrying the empty vector (Figure 8A).

When we cloned *tcdE* in the expression plasmid (see Materials and Methods), we took into account the fact that the actual product of *tcdE* is unknown. There are three potential ATG start codons in the same reading frame (Figure 7B). The most upstream start codon (Met_1_) is located 122 bp downstream of the stop codon of the *tcdB* gene. The second and third potential initiator methionine codons correspond to residues 25 (Met_25_) and 27 (Met_27_) of the putative full-length protein (Figure 7B). Thus, sequences encoding all three potential forms of TcdE (*tcdEM1*) or only M25 and M27 (*tcdEM25*) or only M27 (*tcdEM27*), were cloned under the control of the λ P_R′_ promoter (Figure 7A) [29]. When expressed in *E. coli* MC1063 λ (cI_857_Sam7), the TcdEM1 (pCD311) and TcdEM25 (pCD302) forms induced complete bacterial lysis within five minutes after thermo-induction whereas expression of the TcdEM27 form (pCD322) caused complete lysis only at about 40 min. (Figure 8A). As was the case for λS protein, lysis induced by expression of any of the putative forms of TcdE required expression of the λ endolysin. However, when expressed in *E. coli* λCmrΔ(SR), carrying a deletion in holin and endolysin genes, the λS, TcdEM1, TcdME25 and TcdEM27 forms did not cause lysis (Figure 8B), indicating that the lysis in *E. coli* could not be due to over-expression of the λS and TcdE forms. It has been shown that an inactive mutated version of ‘S’ protein expressed in *E. coli* MC1063 λ (cI_857_Sam7) does not induce host lysis as well [30]. To assess the state of the cytoplasmic membrane in endolysin-deficient cells, we stained the cells with SYTO and PI as described above. Figure 8D shows the plots of green (FL1) and red (FL2) fluorescence of *E. coli* strains after staining with SYTO and PI dyes before and after thermoinduction. Before thermoinduction, the lysogens appeared to have intact cell membranes. The S^105^-expressing *E. coli* λ (CmrΔSR) population lost its membrane integrity nearly 30 minutes after thermoinduction. In TcdEM1 expressing *E. coli* cells, the membrane was permeabilized within five minutes after thermoinduction, indicating a more rapid damage to the cell membrane (Figure 8D).

### Alternative translational motifs in the *tcdE* gene

The λS gene encodes two proteins with opposing functions. The lethal holin S^105^ initiates at codon 3 and its inhibitor, S^107^, initiates at codon 1. The proportion of S^105^ to S^107^, normally 2∶1, is determined by an RNA stem-loop structure that includes the ribosome binding site of λS. This dual start motif appears to be a fine-tuning system for the scheduling of host cell lysis during phage infection [29]. Since we observed a significant difference in the timing of lysis mediated by different TcdE forms in *E. coli* (Figure 8A), we tested the possible roles of the three potential translational start sites in *tcdE* mRNA by introducing site-directed mutations within the putative start codons. We deleted the λS ribosome binding site, creating the plasmid pBRQ(Δrbs) and then cloned *tcdE* ORF from first methionine codon with its own ribosome binding site (*tcdEMet1*) in pBRQ(Δrbs), creating plasmid pRG32 (Figure 7B). When expressed in *E. coli* MC1063 λ (cI_857_Sam7), the TcdEMet1 (pRG32) form induced late bacterial lysis (Figure 8C) compared to the rapid lysis phenotype of *tcdEM1* expressed from the λS RBS (Figure 8A).

To differentiate the lytic activities of the TcdE derived by initiation at codon 1 from those derived by initiation at codon 25 and 27, we converted methionines M25 and M27 to leucine residues and the protein expressed from this construct pRG35 was designated as TcdEMet1' (Figure 7B). Substitution of methionine by leucine was expected to maintain the hydrophobicity of the protein. Following similar strategies, constructs pRG37 and pRG36 expressing TcdEMet25' and TcdEMet27', respectively, were created by converting methionine M1 into a stop codon and then by changing methionines M27 to L27 in pRG37 and M25 to L25 in pRG36 (Figure 7B, Table 1). The expression of the different TcdE forms (Met1', Met25' and Met27'), was tested by introducing a C-terminal 6xHis Tag into the respective constructs and protein expression was confirmed by Western blotting with anti-His tag antibodies (data not shown). When these versions of TcdE, each of which encodes only a single form of the protein, were expressed in *E. coli* MC1063 λ (cI_857_Sam7), we observed major differences in lysis phenotypes (Figure 8C). The TcdEMet1'- and TcdEMet25'- expressing clones initiated lysis within 15 minutes after induction, whereas the TcdEMet27'- expressing clone showed a much slower decrease in OD_600_ that began at about 25 minutes after induction. Both these results indicate that methionines at codons 1, 25 and 27 can be used as start codons. However, the TcdEMet27' clone induced some lysis but at lesser extent, compared to the two other TcdE forms. Moreover, since the TcdEMet1' and TcdEMet25' clones (neither of which includes methionine 27) induced much more rapid lysis than did TcdEMet1 (which potentially expresses as many as three different TcdE forms) or TcdEMet27', the form of TcdE that initiates at M27 may be both relatively inactive and a potential antiholin analogous to λ S^107^ when multiple forms of TcdE are produced simultaneously.

**Table 1:** Bacterial strains, plasmids and phages used in this study.

Strains/Plasmids/Phages	Description	Sources/References
λCmrΔ(SR)	*stf*::*cat*::*tfa c*I857 Δ(*SR*); replacement of *stf* and *tfa* genes (λ nt 19996–22220) with *cat* gene (36); Δ(*SR*); loss of λ nt 45136–45815	[27]
λcI_857_Sam7		[45]
*E. coli* CA434	Conjugation donor	[40]
*C. difficile* JIR8094	Erythromycin sensitive derivate of *C. difficile* 630 strain	[39]
*C. difficile tcdE* mutant	*C. difficile* JIR8094 with intron insertion within *tcdE* gene	This study
pJN4	pBR322 derivative carrying λS under the control of the late transcription regulatory (LTR) elements of phage λ	[28]
pJN5	pBR322 derivative carrying S^105^ under the control of the late transcription regulatory (LTR) elements of phage λ	[28]
pCD311	pJN4 derivative carrying *tcdE*M1 by replacing λS	This study
pCD302	pJN4 derivative carrying *tcdE*M25 by replacing λS	This study
pCD322	pJN4 derivative carrying *tcdE*M27 after replacing λS	This study
pMTL007	Clostron Plasmid	[24]
pMTL007::Cdi-*tcdE*-234a	pMTL007 carrying *tcdE* specific intron	This study
pCD463	pJN4 derivative carrying *tcdE*M_1 -_6His	This study
pBRQ(Δrbs)	pJN4 carrying deletion in λS ribosomal binding site	This study
pRG32	pBRQ(Δrbs) with *tcdE*Met1 with its own ribosomal binding site	This study
pRG35	pRG32 derivative with *tcdE*Met1 carrying M25 and M27 mutated to L25 and L27 respectively to express TcdEMet1'	This study
pRG36	pRG32 derivative with *tcdE*(M1) carrying M1 and M25 mutated into stop codon and L25 respectively to express TcdEMet27'	This study
pRG37	pRG32 derivative with *tcdE*(M1) carrying M1 and M27 mutated to stop codon and L27 respectively to express TcdE Met25'	This study
pRG46	pMTL84151 carrying *tcdE*-6His with 600 bp of its own upstream region	This study
pRFP185	Tetracyclin inducible expression vector for *C. difficile*	[25]
pRG60	pRFP185 carrying *tcdE*-6His with own RBS under tetracyclin inducible promoter	This study

### Localization of TcdE in *E. coli* and *C. difficile*

**Figure 9:**
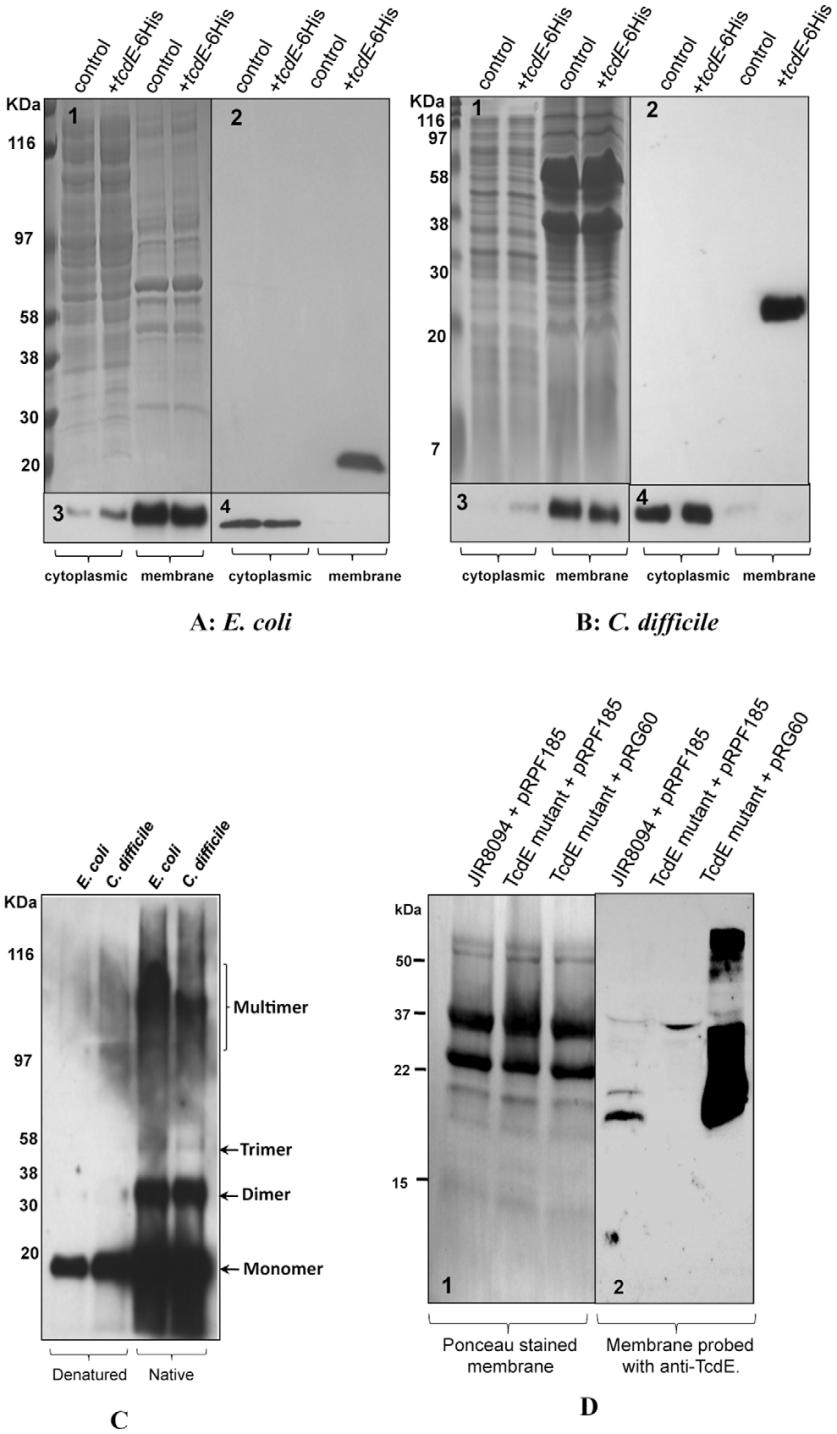
Localization of TcdE in *E. coli* and *C. difficile*. **A.** Cytoplasmic and membrane proteins analysis of *E. coli* lysogens of λCmrΔ(SR) carrying pBR322 (control) or pCD463 (+*tcdE*-6xHis). **B.** Cytoplasmic and membrane proteins analysis of *C. difficile* strain carrying either pMTL84151 (control) or pRG46 (+*tcdE*-6xHis). (1) SDS-PAGE coomassie stained gel. Western blots probed with 6XHis Tag antibody (2), ATPase Beta subunit antibody (3), and Ribosomal subunits LI/L2 monoclonal antibody (4). **C.** Membrane protein samples from bacterial cells expressing TcdE-6His resuspended in denature or native sample buffers and analyzed by Western blot using His-Tag antibody. **D.** Membrane proteins of JIR8094, TcdE mutant and complemented TcdE mutant strains were harvested from bacterial cultures induced with 20 ng/ml of ATc for 2 hours, separated in 16% Tris-Glycine gel and transferred into PVDF membrane. Panels 1. Ponceau stained membrane; 2. Probed with TcdE antibody.

In *E. coli*, the λS protein exists in an oligomeric state in the inner membrane [31]. To localize TcdE, we expressed TcdE with a C-terminal 6xHis Tag (pCD463) in *E. coli* strain MC1063 λCmrΔ(SR), which does not express holin or endolysin (see Materials and Methods). This version of TcdE complemented the λ (cI_857_Sam7) lysogen (Figure 8C). After induction of TcdE expression, cells were collected and the cytosolic and the inner membrane proteins were separated by SDS-PAGE and analyzed by Western blot with antibodies against the 6xHis Tag or ribosomal proteins L7/L12 or the membrane-bound β subunit of *E. coli* ATP synthase (Figure 9A). As expected, the ribosomal subunits were detected only in the cytoplasmic fraction and the ATPase β subunit was detected primarily in the membrane fraction. The anti-6xHis Tag antibodies detected TcdE only in the membrane fraction (Figure 9A), indicating that TcdE is exclusively membrane-bound in *E. coli*.

To localize TcdE in *C. difficile*, we expressed TcdE with a C-terminal 6xHis Tag in a replicative plasmid under the control of the native upstream region (pRG46, see Materials and Methods). Cytosolic and membrane proteins were extracted from the transconjugant and were analyzed by Western blots. The ribosomal proteins L7/L12 were detected only in the cytosolic fractions and the ATPase β subunit only in the membrane fractions (Figure 9B). The anti-6xHis Tag antibodies detected TcdE-6xHis only in the membrane fractions, as was the case in *E. coli* expressing TcdE. Finally, under non-reducing conditions, i.e. in absence of the reducing agent, we observed that TcdE forms oligomers both in *E. coli* and in *C. difficile* (Figure 9C), which is consistent with the status of membrane-associated holins, such as λ holin S^105^ when they are forming holes [31]. We also analysed the membrane proteins of the complemented TcdE mutant by Western blot using antibodies against TcdE, to evaluate the level of TcdE in JIR8094 strain vs. TcdE complemented strain that we have shown secreting more toxins than the parental strain (Figure 4B). Interestingly, the level of TcdE expressed is higher in the complemented strain than in the wild type strain (Figure 9D). Hence the higher amount of toxin in the supernatant of the complemented *tcdE* mutant could be due to the higher level of TcdE in the complemented strain.

## Discussion

A role for holins in protein secretion in Gram-positive bacteria has long been suggested without strong experimental evidence [20]–[22]. We provide here the first genetic evidence that TcdE, an apparent holin, is required for efficient toxin secretion by *C. difficile*, but that it does so without causing the significant cell lysis or membrane damage usually associated with the phage holins. At the same time, we showed that, when expressed in *E. coli*, TcdE has properties similar to those of λ S protein, a holin needed for phage release. Thus, any mechanistic model for the role of TcdE in *C. difficile* toxin secretion has to account for TcdE's inability to cause detectable membrane damage in the natural host. Such models are suggested below.

Holins are extremely diverse, but most can be grouped into two main classes based on the number of predicted transmembrane domains (TMDs) [32]. Analysis of TcdE using the TMHMM program (http://www.cbs.dtu.dk/services/TMHMM) suggested the presence of three TMDs, characteristic of class I holins [32] (Figure 1A). Similar to many class I holins, TcdE expressed in *E. coli* and *C. difficile* was localized to the cytoplasmic membrane in oligomeric forms. Close homologs of TcdE in *Clostridium sordellii* (TcsE), and *Clostridium perfringens* (UviB) could be identified in close proximity to toxin-encoding genes. We expressed these TcsE and UviB downstream of the λ late promoter in *E. coli* MC1063 λ (cI_857_Sam7). Similar to TcdE, both TcsE and UviB induced cell lysis upon phage induction (data not shown). Mutagenesis of the genes encoding these TcdE-like proteins will indicate if that they are required for secretion of the lethal toxin of *C. sordellii* and the bacteriocin of *C. perfringens*.

Holin-encoding genes and holin activity are subject to several levels of regulation, among which a particularly striking feature is the common occurrence of two potential translational starts separated by only a few codons [18], [33]. The TcdE coding sequence includes three potential start codons at positions 1, 25 and 27. We have tested all three potential TcdE forms (TcdEM1, TcdEM25 and TcdEM27) for their ability to complement a λS mutant phage. The M1 and M25 forms of TcdE induced lysis within a few minutes after phage induction, whereas the M27 form was only able to induce delayed and less abundant lysis. Constructs that permitted simultaneous expression of M27 and M25 or all three forms showed relatively delayed lysis, raising the possibility that M27 is an inhibitory form of TcdE. Detailed experiments are in progress to test the multi-start regulation of TcdE activity in *C. difficile*.

With TcdE having both structural and biochemical properties of phage holins, we propose several models for the TcdE-dependence of *C. difficile* toxin secretion. Some holins create pores that are wider than 1 µm [34], while others, called pinholins, make channels only 15 Å in diameter [35]. Detergent-solublized λS^105^ holin forms ring-like structures containing about 72 monomers with an average inner diameter of 8 nm [36]. If toxins are secreted in an unfolded state, possibly via translationally coupled secretion, only a narrow channel in the cytoplasmic membrane would be needed. Such a channel would not allow cytoplasmic protein leakage. On the other hand, if toxins are secreted as fully folded proteins, a large membrane channel would be needed due to the volumes the large toxin proteins would occupy. Although TcdE has the intrinsic ability to form pores in the membrane that lead to permeability and cell death, as seen in *E. coli*, it does not do so in *C. difficile*. If TcdE-dependent pores are formed in *C. difficile*, they should be tightly regulated by a mechanism that could include the toxins themselves. The toxins could, for instance, act as plugs in the TcdE pore to prevent loss of solutes or proteins from the cells. Such a model is consistent with our observation that a *tcdA tcdB* double mutant lysed more rapidly than the parental and PaLoc negative strains (Figure 6A). By expressing TcdE at a lower level, *C. difficile* may also efficiently control cell lysis. We were unable to complement the *tcdE* mutant using a wild-type gene on a multicopy plasmid, which suggested that TcdE becomes lethal to *C. difficile* above a certain threshold concentration. This became more evident when we succeeded in complementing the TcdE mutant using a controlled expression vector. Hyper induction driven by high concentrations of the inducer (>50 ng/ml) and prolonged induction of TcdE in *C. difficile* affected the bacterial growth (Figure 4A and data not shown). Hence under natural conditions *C. difficile* presumably expresses an amount of TcdE sufficient to form pores that allow release of toxin without causing cell lysis.

Finally, TcdE-dependent channels might be formed in association with other proteins that control the opening of the pore or TcdE could form a specific, gated channel that only opens in the presence of TcdA/TcdB, without inducing cell lysis. None of these models are mutually exclusive; multiple mechanisms may contribute to toxin secretion without inducing cell lysis in *C. difficile*. Physiologically, it is also more appropriate for *C. difficile* to release its toxins without killing itself, since the goal of toxin secretion is presumably to increase the survival and dispersion of *C. difficile*. The detailed, mechanistic understanding of the holin-dependent systems that mediate toxin secretion by *Clostridium* spp. will be an important advance in understanding how Gram-positive pathogens efficiently release important proteins outside the cell in the absence of known secretion and export signals.

Recently, Olling and collaborators [37] concluded that toxin release was correlated with bacteriolysis, but not with TcdE expression. These results are in direct contradiction with those presented here. Whereas this discrepancy might be attributable to differences in experimental conditions used by the two groups (e.g., glucose-containing BHI medium vs. TY), they are more likely due to differences between the two parental strains used and differences in the time points used for analysis. We have measured the effect of TcdE in late exponential and early stationary phase, whereas the analysis of Olling et al. was primarily restricted to very late time points. Although both strains are erythromycin-sensitive derivatives of strain 630, they were isolated independently and have been maintained through multiple sub-cultures. As discovered by N. Minton and coll. (personal communication), the two extant Erm^s^ 630 strains have acquired a number of divergent mutations, some of which might obscure evidence of the role of TcdE in toxin secretion at late exponential growth phase. Contrasting results with these strains were also seen in studies of the roles of the individual toxins in *C. difficile* pathogenesis [26], [38]. We also note that Olling et al. reported two independent *tcdE* mutants that behaved somewhat differently, suggesting that at least one of the strains had acquired an additional mutation(s).

## Materials and Methods

### Bacterial strains and growth conditions

*C. difficile* strains JIR8094 [39] and its *tcdE* mutant as well as *C. difficile* strains 630 delta *erm* and its A^−^B^−^ mutant [26], were grown anaerobically (10% H_2_, 10% CO_2_ and 80% N_2_) in TY broth or TY agar as described previously [8]. *E. coli* strain HB101 (pRK24) used for conjugation was cultured aerobically in 2×YT. When necessary, *E. coli* cultures were supplemented with chloramphenicol or ampicillin, at 30 µg ml^−1^ and 100 µg ml^−1^, respectively. All routine plasmid constructions were carried out using standard procedures.

### Construction of a *tcdE* knockout mutant

The *tcdE* mutant was generated in *C. difficile* JIR8094 by insertion of the bacterial group II intron using the ClosTron gene knockout system as described by Heap et al. [24]. The insertion site, in antisense orientation between nucleotides 234–235 of the *tcdE* ORF, was selected to design intron-retargeting primers (Table 2). Plasmid retargeting was carried out as described [24]. The resulting plasmid, pTUM007:Cdi-*tcdE*-234a, was transferred to *C. difficile* JIR8094 strain by conjugation as described previously [40]. Thiamphenicol-resistant transconjugants were resuspended in 200 µl of TY broth and plated on TY agar plates containing erythromycin (5 µg ml^−1^) to select potential Ll.ltrB insertions. Then the putative *tcdE* mutants were screened by PCR using *tcdE*-specific primers (OBD231, OBD232) in combination with the EBS-U universal and ERM primers (Table 2). The selected mutant was further characterized by sequencing PCR products amplified using ODB231-232 and EBS-U, and designated strain JIR8094 *tcdE* mutant.

**Table 2:** Oligonucleotides used in this study.

*Name*	*Sequence 5′ to 3′*
OBD231	GGTGGACTATTCTAGATGCACAGTAGTTC
OBD232	TTTGTTAAAAGCTTTATTATATCTACC
OBD256	ACCTAGGAGTCTAGATGAATATGACAATAT
OBD294	GGAGCGTTATCTAGATGACAATAT
OBD 418	GGTGGACTATTCTAGATGCACAGTAGTTC
OBD419	CCCGGGCTTTTCATCCTTAGCATTCAT
OBD444	GCATGCAAAAAAGCATGCAAAGGTATTAATTTAAT
OBD445	TGCGCATTAATGATGATGATGATGATGCTTTTCATCCTTAGCATT
OBD488	TCTAGATGCACAGTACTTCACCTTTT
OBD490	CGCCAGTAAGCTTGAATTCGCCCTTTT
IBS	AAAAAAGCTTATAATTATCCTTAAATATCCATGCTGTGCGCCCAGATAGGGTG
EBS-1	CAGATTGTACAAATGTGGTGATAACAGATAAGTCCATGCTATTAACTTACCTTTCTTTGT
EBS-2	TGAACGCAAGTTTCTAATTTCGGTTATATTCCGATAGAGGAAAGTGTCT
EBS-U	CGAAATTAGAAACTTGCGTTCAGTAAAC
ERM	ACG CGTGCGACTCATAGAATTATTTCCTCCCG
ORGE	TAAGACATCTAGATAAAAAGGTGGACTATGATGA
ORG102	GAG CTC CAATAA AAA GGT GGA CTATGA TGA
ORG103	GGATCC TTA ATG ATG ATG ATG ATG ATG CTT TTC ATC CTT AGC

#### Southern hybridization

Genomic *C. difficile* DNA were digested with *Eco*RV and *Hin*dIII and subjected to agarose gel electrophoresis (0.8%). The DNA were then transferred from the gel onto Hybond-N+ filter (Amersham) in 20X saline citrate according to the method of Southern. Filter was prehybridized for 2 h at 42°C in 50% formamide, 5X SSC, 2x Denhart's solution and 100 mg of denatured salmon sperm DNA per ml. Overnight hybridization was carried out in the same solution at 42°C with a nick-translated intron fragment (OBD522–OBD523, Table 2). The filter was washed 30 min in 1×SSC, 0,1%SDS and 30 min in 0.1×SSC, 0,1%SDS at room temperature before it was air dried and exposed to Amersham Hyperfilm MP.

### *C. difficile* toxin and Lactate dehydrogenase (LDH) assays

Culture supernatants were collected and filtered, and the cell pellets were resuspended in 10 mM Tris buffer, pH 8.0 containing a protease inhibitor cocktail (Roche, Mannheim, Germany). The cytosolic contents were obtained by sonication of the cells, followed by brief centrifugation to removed unbroken cells and cell debris. Total protein concentration was determined using the Bio-Rad protein assay reagent. Equal amounts of cytosolic and supernatant proteins were assayed for their relative toxin contents using the ELISA kit following the manufacturer's directions. LDH activity was determined using the CytoTox 96 kit from Promega (Madison, WI, USA) and toxins were measured using the Premier Toxin A&B Enzyme linked Immunoassay (ELISA) kit from Meridian Diagnostics Inc., [Cincinnati, Ohio].

### Dot blots to detect Toxin A, Toxin B, RNA polymerase and ribosomal subunits

Culture supernatants collected from the parent and the mutant strains were concentrated by passage through Amicon Microcon (YM-100) columns. The cytosolic proteins were prepared by sonication followed by brief centrifugation. To detect TcdA and TcdB, 200 ng of total protein was spotted on nitrocellulose filters (Amersham Pharmacia) and probed with monoclonal antibodies raised against Toxin A [41] and Toxin B (a generous gift from Dr. Feng, University of Maryland). For other blots, two-fold dilutions of *C. difficile* cytosolic proteins were spotted and probed with monoclonal antibodies raised against the *E. coli* RNA polymerase beta subunit (NeoClone Biotechnology) or streptococcal L7/L12 ribosomal subunits [42]. Following incubation with anti-mouse horseradish peroxidase-conjugated antibody, the antigen-antibody complexes were detected using ECL Western blotting detection reagents (Pierce).

### FACS analysis

Bacterial cells were washed with 0.9% NaCl and stained with SYTO9 and Propidium Idodide (PI), mixed in equal proportions, for 5 min. Overnight cultures of *C. difficile* were stained under anaerobic conditions. The thermo-induced *E. coli* cells were chilled on ice before processing for staining. Flow cytometry was performed immediately after staining with a FACS Calibur (Becton Dickinson, San Jose, Calif.) equipped with an air-cooled 15-mW argon ion laser operating at 488 nm. The green fluorescence of the SYTO dye (FL1) was collected using a 530- ±30-nm band-pass filter; the red fluorescence emitted from PI (FL2 or FL3) was collected using a 630- ±10-nm band-pass filter. Control samples were used for the instrument settings (voltage of the detectors and the compensation) and consisted of unlabeled cells, heat-killed bacterial cells stained with PI (FL3 or FL2), and SYTO (FL1). Bacterial cells were discriminated from electronic noise using a double threshold set on both side scatter (SSC) and forward scatter (FSC), with FSC set on E01 and SSC set on 400V. The data were analyzed with the CellQuest software from Becton Dickinson. All parameters were measured using logarithmic amplification.

### Complementation of *C. difficile tcdE* mutant

The *tcdE* ORF along with its RBS was PCR amplified from JIR8094 chromosomal DNA using primers ORG102 and ORG103, which carried restriction sites *SacI* and *BamHI*, respectively. The resulted PCR product was digested with *SacI* and *BamHI* enzymes and cloned into the vector pRPF185 [25], placing the *tcdE* gene under a tetracycline inducible promoter. The resulting plasmid pRG60, was then introduced into JIR8094 and *tcdE* mutant *C. difficile* strains by conjugation. Transconjugants carrying either pRG60 or the vector pRPF185 were grown overnight in TY medium supplemented with thiamphenicol. 100 ml of fresh cultures were inoculated with 1 ml of overnight cultures and were grown for 4 hours in TY medium with thioamphenicol before to be induction with 20 ng/ml ATc. Culture supernatants were harvested for the detection of released toxins using ELISA or dot blot analysis using monoclonal antibody against TcdA.

### TcdE expression under the control of phage λ regulatory elements

Plasmids pJN4 and pJN5, derivatives of pBR322 that carry the λ genes of Sλ (encoding holin *S^105^* and antiholin *S^107^*) or *S^105^*, respectively, were used to create constructs in which the *tcdE* gene was placed under the control of the λ pR′ promoter (late transcription regulatory (LTR) region, spanning from the 3′ end of the antiterminator Q gene to the first bp of the S holin gene) [28]. Three different forms of the *tcdE* coding sequence, known as *tcdEM1*, *tcdEM25* and *tcdEM27* (Figure 6B), were PCR amplified using forward primers OBD231, OBD256 and OBD294, respectively (Table 2) along with the reverse primer OBD232, digested and ligated to pJN5 to create plasmids pCD311 (*tcdEMI*), pCD302 (*tcdEM25*) and pCD322 (*tcdEM27*). Using site-directed mutagenesis ((Stratagene Quick change mutagenesis kit), the Sλ gene ribosomal binding site (RBS) was deleted in plasmid pJN4 to create plasmid pBRQ(Δrbs). To express TcdE with its own ribosomal binding site, the *tcdEM1* gene was PCR amplified using primers ORGE1 and OBD232 and the product (*tcdEMet1*) was cloned into pBRQ(Δrbs), creating plasmid pRG32. Site-directed mutagenesis was performed in pRG32 to introduce mutations in the TcdE coding regions and resulted in plasmids pRG35, pRG36 and pRG37 (Table 1, Figure 6C). In order to test TcdE holin activity, lysogens of *E. coli* strain MC1061 for a defective λ prophage bearing a nonsense mutation in its holin gene (Sam7) or carrying a deletion in holin and endolysin genes [λCmrΔ(SR)] were used as hosts for these plasmid constructs. Both λ (Sam7) and λCmrΔ(SR) encode a thermo-sensitive CI repressor (cI_857_) and are induced upon shifting the culture temperature from 30°C to 37°C [27]. Resultant strains were grown in LB broth at 30°C until the OD_600_ reached 0.15–0.2 before thermo-induction of the λ prophage at 42°C for 15 min. Bacterial growth and lysis at 37°C were then followed by monitoring the absorbance at 600 nm at 5 min intervals.

### Localization of TcdE in *E. coli*

To fuse a 6xHis Tag to the C-terminus of TcdEM1, the *tcdEM1* was amplified with primer OBD418 and OBD419 (Table 2), digested and cloned in pIVEX2.3d (Roche Applied Science) to create pCD429. Then, a 6xHis Tag fused to *tcdEM1* was amplified from pCD429 DNA using OBD488 and OBD490 (Table 2), digested and ligated to pJN5 creating plasmid pCD463. The plasmids pCD463 and pBR322 were introduced into *E. coli* strain MC1061:: λCmrΔ(SR) and the cultures were grown in LB broth at 30°C until the OD_600_ reached 0.8–1.0 before the thermo induction (15 min at 42°C) and was then kept at 37°C for two more hours. The cells were then harvested by centrifugation, resuspended in 0.05 M Tris-HCl, pH 7.5, containing a protease inhibitor cocktail (Sigma), and disrupted by passage through a French pressure cell at 10000 psi. The inner membrane and the cytosolic proteins were prepared as described [29]. Equal amounts of inner membrane and cytosolic proteins were separated on a SDS-PAGE gel and Western blots were performed with antibodies against the 6xHis Tag (Bio-Design International), *E. coli* ATPase β subunit [43] and streptococcal ribosomal proteins L7 and L12 [42].

### Localization of TcdE in *C. difficile*

The *tcdE* gene, along with 600 bp of the upstream region, was amplified with primers OBD444 and OBD445 introducing a C-terminal 6xHis Tag (Table 2). The PCR product was cloned in the clostridial conjugative vector pMTL84151 to create plasmid pRG46, which was introduced by conjugation into *C. difficile* strain N04-799 (National Microbiology Laboratory, Manitoba, Canada), a ribotype 027 strain. Transconjugants carrying either pRG46 or the vector pMTL84151 alone were grown overnight in TY medium supplemented with thiamphenicol. Cytosolic and membrane proteins were prepared as described before [44]. Membrane protein samples from bacterial cells expressing TcdE-6His were denatured by resuspending them in sample buffer with DTT and were boiled for 5 minutes before loading into 4–20% gradient Tris-Glycine gel. Under non-reducing conditions, membrane proteins were resuspended in sample buffer in the absence of the reducing agent (DTT) and were not boiled before loading into the gel. After separation the proteins were transferred into PVDF membrane and were probed with antibodies against the 6-His Tag or TcdE as described earlier [37].

## Supporting Information

Figure S1 Comparative analysis of toxin B production by JIR8094 and *tcdE* mutant strains. African green monkey kidney (Vero) cells, were cultured in Dulbecco's modified Eagle's medium (DMEM, Gibco) supplemented with 5% fetal calf serum (PAA), 50 U/ml penicillin and 50 µg/ml streptomycin (Gibco) at 37°C in a 5% CO_2_ atmosphere. Cells were grown until confluence in 96-well plates. Supernatants from 12 hours old bacterial cultures were used for the cytotoxicity assay. The monolayers were incubated with 2-fold serially diluted in DMEM of supernatants. After 24 h at 37°C, cytotoxicity was assessed by examination using an optical microscope. A positive result was considered when more than 50% of cells showed a cytotoxic effect (characteristic rounding of Vero cells). The data shown are the mean +/− standard error of three replicative samples.
(TIF)
