# Supplementary figures and images for: Secretion of Clostridium difficile Toxins A and B Requires the Holin-like Protein TcdE
Source: PLoS Pathog. 2012 Jun 7;8(6):e1002727. doi: 10.1371/journal.ppat.1002727 (PMC3369941; doi:10.1371/journal.ppat.1002727)

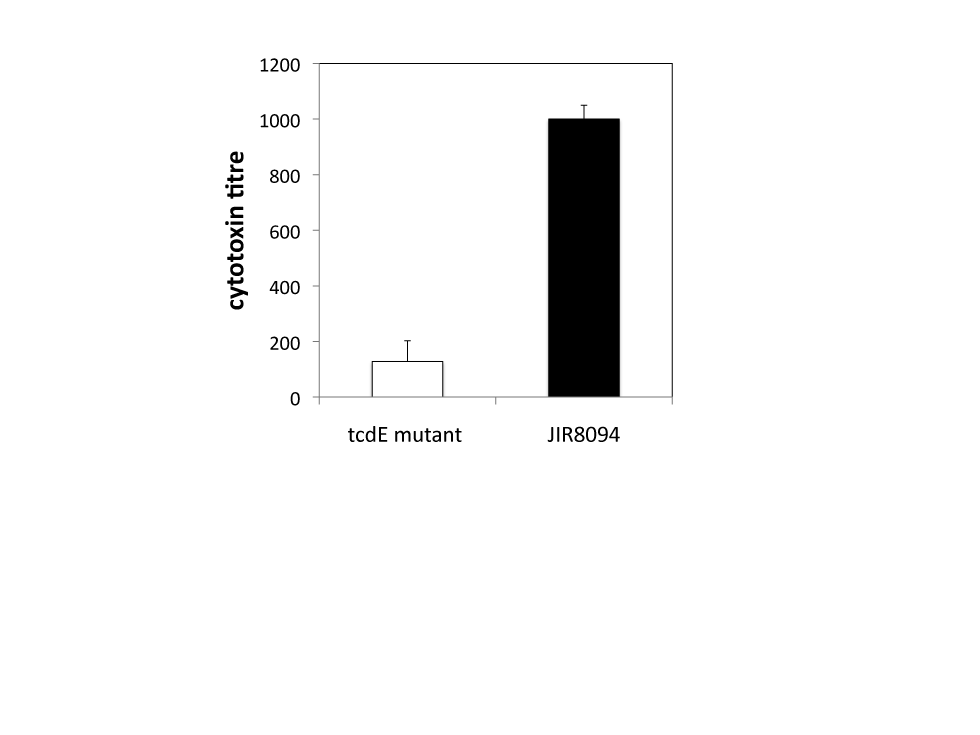

Supplement: Figure S1 — Comparative analysis of toxin B production by JIR8094 and tcdE mutant strains. African green monkey kidney (Vero) cells, were cultured in Dulbecco's modified Eagle's medium (DMEM, Gibco) supplemented with 5% fetal calf serum (PAA), 50 U/ml penicillin and 50 µg/ml streptomycin (Gibco) at 37°C in a 5% CO2 atmosphere. Cells were grown until confluence in 96-well plates. Supernatants from 12 hours old bacterial cultures were used for the cytotoxicity assay. The monolayers were incubated with 2-fold serially diluted in DMEM of supernatants. After 24 h at 37°C, cytotoxicity was assessed by examination using an optical microscope. A positive result was considered when more than 50% of cells showed a cytotoxic effect (characteristic rounding of Vero cells). The data shown are the mean +/− standard error of three replicative samples. (TIF) [file ppat.1002727.s001.tif]
